# Extrapulmonary tuberculosis in Africa: Molecular analysis of clinical specimens of suspected cases in Northern Ghana

**DOI:** 10.1002/puh2.160

**Published:** 2024-02-14

**Authors:** Yaa Nyarko Addai, Samuel E. K. Acquah, Honesty Mensah Ganu, Ezekiel Kofi Vicar, David Zeyeh, Abass Abdul Karim, Walana Williams, Israel Mensah Attipoe, Lawrence Quaye

**Affiliations:** ^1^ Department of Biomedical Laboratory Sciences School of Allied Health Sciences University for Development Studies Tamale Ghana; ^2^ Department of Infectious Diseases School of Allied Health Science University for Development Studies Tamale Ghana; ^3^ Chest Clinic Korle‐Bu Teaching Hospital Accra Ghana; ^4^ Department of Clinical Microbiology School of Medicine University for Development Studies Tamale Ghana; ^5^ Department of Microbiology Tamale Teaching Hospital Tamale Ghana; ^6^ Public Health Laboratory Ghana Health Service Tamale Ghana

**Keywords:** extrapulmonary tuberculosis, line probe assay, *Mycobacterium tuberculosis*, Tamale Teaching Hospital

## Abstract

**Background:**

Extrapulmonary tuberculosis (EPTB) is a major component of the total tuberculosis cases reported by the World Health Organization. This is a study conducted to compare microscopy and molecular techniques to determine the prevalence of *Mycobacterium tuberculosis* complex (MTBC) in EPTB patients.

**Methods:**

Smear microscopy and genotype MTBDR*plus* line probe assay (LiPA) were applied to concentrated extrapulmonary clinical specimens from different anatomic sites to determine the presence of *M. tuberculosis* and their susceptibility to isoniazid (INH) and/or rifampin (RIF).

**Results:**

A total of 251 specimens comprising 108 (43%) ascitic fluid, 54 (21.5%) pleural aspirate, 24 (9.6%) gastric lavage, 15 (6.0%) pus, 9 (3.6%) synovial fluid, 5 (2%) cerebrospinal fluid, 2 (0.8%) breast aspirate and 34 (13.5%) aspirates from unindicated sites obtained from patients with suspected EPTB attending the Tamale Teaching Hospital were analysed. Microscopically, acid fast bacilli (AFB) were detected in one ascitic fluid and a pus specimen. Using the LiPA, MTBC was observed in four (2.6%) samples; three (3) ascitic fluid and one aspirate.

**Conclusion:**

*M. tuberculosis* complex was confirmed in four (2.6%) patients. The most common specimens suspected of EPTB were ascitic fluid, pleural aspirate and gastric lavage. However, MTBC was predominantly detected in ascitic fluid. This result indicates that the LiPA can improve the detection of EPTB in the region and similar settings globally.

## Graphical abstract

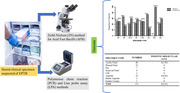

## INTRODUCTION

Tuberculosis (TB) remains one of the leading causes of morbidity and mortality worldwide. The World Health Organization (WHO) estimates that 10.6 million people fell ill with TB in 2021 out of which 1.6 million died [1]. The disease mostly affects the lungs but can affect other anatomical sites. Pulmonary TB involving lung parenchyma is the widely reported case of *Mycobacterium tuberculosis* disease representing 75% of all TB cases. Studies have documented the emergence of extrapulmonary tuberculosis (EPTB) as an important public health problem worldwide, especially in low‐income communities. The prevalence of EPTB cases reported globally in 2018 was 15% and Africa reported 16% among all TB cases. The EPTB report for Ghana, however, was 8% [2, 3].

Risk factors associated with EPTB include immunosuppression, human immunodeficiency virus (HIV) infection, gender and age [4, 5, 6, 7]. Approximately 33%–50% of people living with HIV (PLHIV) with latent tuberculosis are more likely to develop EPTB [8]. Although reports suggest male gender as a risk factors [6, 6], other studies have found females and increasing age to be more associated with EPTB [6, 9–11]. In immunosuppressed patients, EPTB is most likely to originate from a primary focus in the lung tissue or may be seeded in miliary TB and spread through lymphatic or haematogenic route to other organs or sites of the body such as pleura, pericardia, lymph nodes, gastrointestinal tract, urinary tract, skin, joints, bones and meninges [12, 13]. TB pleurisy, pleural infusion, pericardial effusion and lymphadenitis have been reported in 30%–95% of individuals with HIV/AIDS in developing countries [14]. Immunocompetent individuals acquire EPTB mainly through the consumption of contaminated meat and meat products, unpasteurized milk and milk products from infected livestock [15, 16].

EPTB is characterized by insidious, chronic and clinically nonspecific symptoms. Diagnosis of EPTB is generally challenging and often delayed due to the wide differential diagnosis, limited accessibility to the affected sites, paucibacillary load in the clinical specimen and low performance characteristics of conventional diagnostic assays. Novel diagnostic methods such as nucleic acid amplification tests are recommended as the preferred assays for effective diagnosis of EPTB.

The current study applied the MTBDR*plus* line‐probe assay (Hain Lifescience GmbH) to determine the prevalence of *M. tuberculosis* complex (MTBC) and their susceptibility to rifampin (RIF) and isoniazid (INH) in EPTB patients.

## METHODS

### Study design, setting and population

We conducted this cross‐sectional study among patients suspected of EPTB attending the Chest Clinic of the Tamale Teaching Hospital (TTH). The TTH is a tertiary healthcare facility, which serves as a referral facility for the five regions of Northern Ghana. The hospital offers all the four classes of services: curative, preventive, promotive and rehabilitative care. The Chest Clinic is a specialist clinic of the hospital that attends to in‐ and outpatients with diseases caused by *M. tuberculosis*. All suspected causes of pulmonary infections are directed to this clinic for care. In addition, all cases of TB are referred to the Chest Clinic. In this study, all cases referred to the laboratory for EPTB investigation which had well‐labelled pathological number, constituted the study population and served as the sample size.

### Specimen, variable and data collection

Stored specimens obtained from organs or anatomical sites other than lung parenchyma of patients with suspected EPTB were retrieved from the refrigerator in the bacteriology laboratory of the TTH. By reverse tracing using the unique specimens’ pathological numbers, patients’ biodata including age and sex, clinical information on wards of patients and anatomical sites of specimens were extracted from the laboratory records. Specimens with no traceable pathological numbers were exempted from the analysis.

### Laboratory methods

Ziehl–Neelsen (ZN) method for acid fast bacilli (AFB), polymerase chain reaction (PCR) and line probe assay (LiPA) was performed by standard methods.

#### Microscopy

The macroscopic characteristics of the specimens which encompass turbidity, the presence of blood and mucus were noted, and 1.0 mL of the specimen spun at 3000 rpm for 15 min in labelled 5 mL test tubes. Three smears were prepared from the sediments on labelled slides. One slide was stained using the ZN method for AFB. The second slide was stained using the fluorochrome (auramine) staining method, whereas the third smear was stained with the Gram staining method. AFB‐positive smears were graded according to the IUALTD/WHO standards as described by Javed et al. [17]. The remaining samples were processed for LiPA using the *N*‐acetyl‐l cysteine–sodium citrate‐NaOH (NALC–NaOH) method as previously described by Kent et al. [18].

DNA extraction using Genolyse. A loop full of the concentrate was suspended in 300 μL molecular grade water and heat killed at 95°C for 60 min in a heating block. The preparation was pelleted for 15 min at 10,000 × g and the pellet suspended in 100 μL Lysis buffer (A‐LYS) by vortexing. The preparation was again incubated for 5 min at 95°C in a heating block and 100 μL neutralization buffer (A‐NB) added. The final preparation was spun down for 5 min at full speed and the supernatant aliquoted for PCR.

#### Line probe assay PCR

Reagents for gene amplification were constituted in reaction mixtures A and B (AM‐A and AM‐B). Regions of the DNA associated with mutations that are most frequently identified in resistant strains conferring resistance to isoniazid and rifampicin were amplified in the presence of 10 μL AM‐A, 35 μL AM‐B and 5 μL DNA for 35 cycles of 95°C for 15 min, 95°C for 30 s, 65°C for 2 min, 95°C for 25 s and 50°C for 40 s, 70°C for 40 s and 70°C for 8 m. All assays were well controlled by including positive controls as well as a negative control (nuclease‐free water). The PCR products were stored at −20°C to be used later for hybridization.

#### Line probe assay hybridization

The PCR products were denatured in a denaturation solution for 5 min at room temperature in hybridization wells. Membrane strips coated with specific probes complementary to the amplified genomic regions were placed into the wells and prewarmed hybridization buffer (45°C) added with gentle shaking on the TwinCubator shaking platform for 30 min at 45°C. Stringent wash solution was then added for 15 min at 45°C and then incubated in diluted streptavidin‐conjugated alkaline phosphatase for 30 min on the shaking platform. Substrate solution was added and incubated at room temperature protected from light without shaking for up to 20 min. The alkaline phosphatase transforms the substrate into a dye which becomes visible on the membrane strips as a coloured precipitate. Only LiPA reaction regions with bands intensities equal or stronger than that of the Amplification control zone were considered positive. Identification of specific drug resistance was based on the absence of specific wild‐type band and/or the presence of mutation band corresponding to specific mutations within the *katG* gene and *inhA* promoters for INH and the *rpoB* gene for RIF. All procedures were performed in different rooms and in accordance with the manufacturer's protocol (Hain Lifescience GmbH).

### Data analysis

Patients’ demographic and clinical data were entered into Excel spreadsheet. Statistical Package for Social Scientists (SPSS) software version 22 (SPSS Inc.) was used to analyse the data. Frequencies/percentages were determined for categorical variables and mean estimated for the continuous variable (age). Cross‐tabulations and sample proportions were used to compare the studied variables.

### Ethical considerations

Approval for the conduct of the study was obtained from the Ethical and Research Committee of the University for Development Studies (UDS/RB/0121/22), and permission from the TTH. Informed consent was waived because of the retrospective nature of the study.

## RESULTS

### Distribution of specimens by patients’ age, gender and ward

**FIGURE 1:**
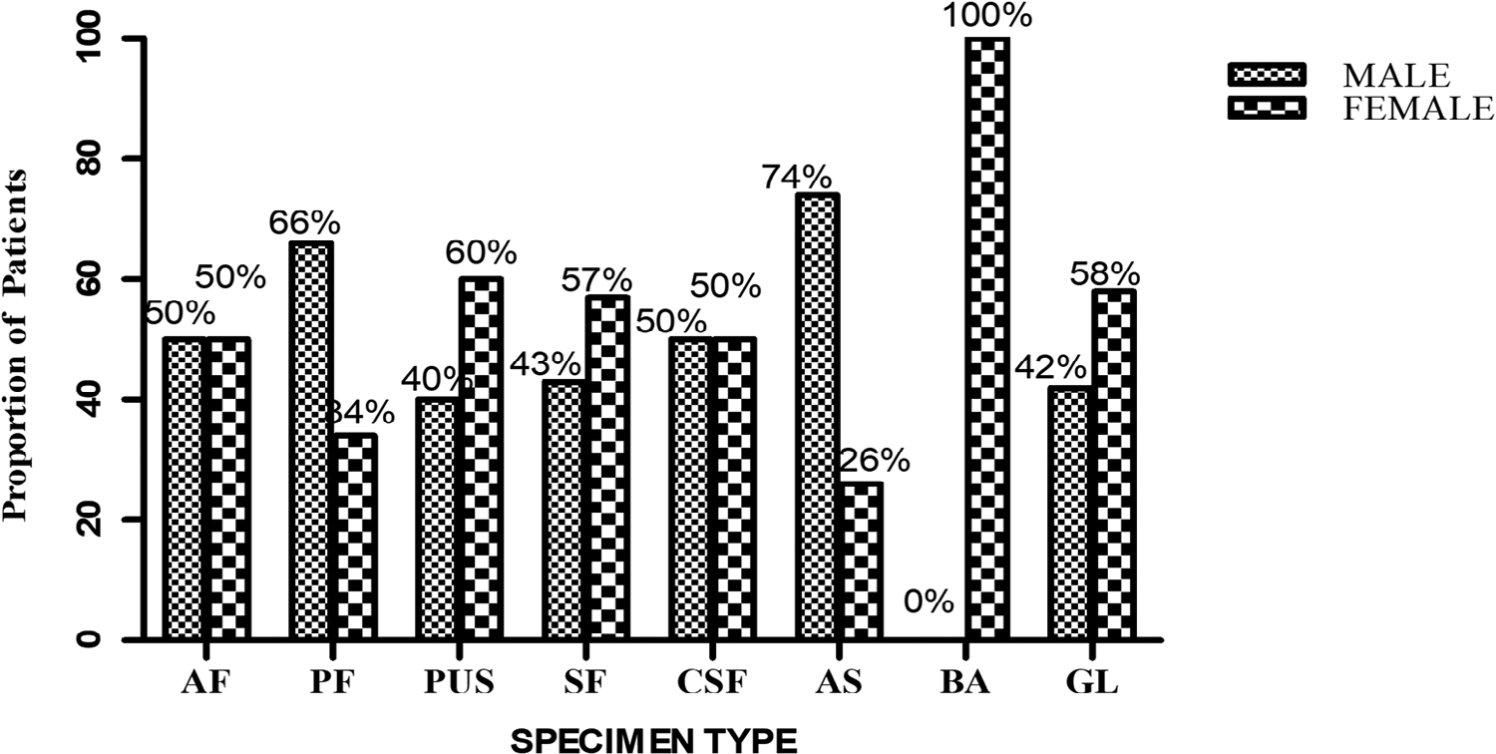
Specimen type and patient gender. AF, ascitic fluid; AS, aspirate; BA, breast aspirate; CSF, cerebrospinal fluid; GL, gastric lavage; PF, pleural fluid; SF, synovial fluid.

In all, 251 specimens obtained from patients suspected of EPTB were examined. The specimen comprised predominantly ascitic fluid 108 (43%), pleural aspirate 54 (21.5%) and gastric lavage 24 (9.6%). The macroscopic assessment of the specimens showed that the majority 111 (44.2%) were turbid. A total of 210 (83.7%) specimens had patients’ gender indicated, comprising 112 (53.3%) males and 98 (46.7%) females (Figure 1).

Patients’ ages were indicated on 196 (78.1%) specimen. The mean age of the patients was 32.3 ± 22.8. The predominant specimen was ascitic fluid which was mostly obtained from patients ≥15 years old, whereas gastric lavage was mostly obtained from patients >15 years old (Table 1). Majority 178 (70.9%) of the specimen were obtained from the medical wards, whereas the chest ward presented the least number of specimens, 1 (0.4%) (Table 1).

**TABLE 1:** Specimen type and general characteristics of suspected extrapulmonary tuberculosis (EPTB) patients who reported to the Tamale Teaching Hospital for diagnosis and treatment.

	Specimen type								
Variables	Ascetic fluid *n* (%)	Pleural fluid *n* (%)	Pus *n* (%)	Synovial fluid *n* (%)	CSF *n* (%)	Aspirate *n* (%)	Breast aspirate *n* (%)	Gastric lavage *n* (%)	Total
Age groups (years) (*N* = 196)									
<15	12 (23.5)	6 (11.8)	5 (9.8)	2 (3.9)	3 (5.9)	1 (2.0)	0 (0.0)	22 (43.1)	51
15–30	28 (54.9)	13 (25.5)	4 (7.8)	2 (3.9)	0 (0.0)	3 (5.9)	0 (0.0)	1 (2.0)	51
31–45	24 (52.2)	10 (21.7)	2 (4.3)	1 (2.2)	1 (2.2)	7 (15.2)	1 (2.2)	0 (0.0)	46
46–60	10 (47.6)	6 (28.6)	1 (4.8)	0 (0.0)	0 (0.0)	3 (14.3)	1 (4.8)	0 (0.0)	21
61–75	9 (47.4)	6 (31.6)	1 (5.3)	2 (10.5)	0 (0.0)	0 (0.0)	0 (0.0)	1 (5.3)	19
>75	2 (25.0)	4 (50.0)	0 (0.0)	0 (0.0)	0 (0.0)	2 (25.0)	0 (0.0)	0 (0.0)	8
Ward (*N* = 251)									
Medical	90 (50.6)	45 (25.3)	4 (2.2)	6 (3.4)	3 (1.7)	28 (15.7)	0 (0.0)	2 (1.1)	178
Surgical	1 (11.1)	1 (11.1)	4 (33.3)	1 (11.1)	1 (11.1)	2 (22.2)	2 (22.2)	0 (0.0)	12
Paed	7 (17.1)	6 (14.6)	4 (9.8)	1 (2.4)	1 (2.4)	0 (0.0)	0 (0.0)	22 (53.7)	41
A/E	6 (50.0)	1 (8.3)	1 (7.6)	1 (8.3)	0 (0.0)	4 (33.3)	0 (0.0)	0 (0.0)	13
Gyn	4 (66.7)	0 (0.0)	2 (33.3)	0 (0.0)	0 (0.0)	0 (0.0)	0 (0.0)	0 (0.0)	6
Chest	0 (0.0)	1 (100.0)	0 (0.0)	0 (0.0)	0 (0.0)	0 (0.0)	0 (0.0)	0 (0.0)	1

Abbreviations: A/E, accident and emergency, Gyn, gynaecology, Paed, paediatrics.

### Outcome of microscopy and LiPA

The three staining methods (Gram, ZN and fluorescence microscopy [FM]) detected bacteria in 14.3% (36/251) of the specimens examined. These comprised 14 (42.4%) Gram‐positive cocci, 12 (36.4%) Gram‐positive rods and 7 (21.2%) Gram‐negative rods. Additionally, 2 (0.8%) AFB and 1 (0.4%) FM bacilli were detected (Table 2). Out of the 251 specimens, 151 (60.2%) were subjected to the LiPA. The assay detected a total of 4 (2.6%) *M. tuberculosis* from 3 (1.98%) ascitic fluid and 1 (0.66%) aspirate specimens (Table 3). Drug resistance was detected in two of the three ascitic fluid isolates; one was an multidrug resistant (MDR) strain, whereas the other was mono‐drug resistant to INH.

**TABLE 2:** Specimen type and microscopic results from extrapulmonary tuberculosis (EPTB) suspected patients.

		Microscopy				
Sample	Number	Gram reaction			Acid fast staining	
		GNR (%)	GPC (%)	GPR (%)	ZN Pos (%)	FM Pos (%)
Ascitic fluid	108	2 (1.9)	2 (1.9)	5 (4.6)	1 (0.9)	1 (0.9)
Pleural fluid	54	4 (7.4)	4 (7.4)	5 (9.3)	0 (0.0)	0 (0.0)
Pus	15	0 (0.0)	2 (13.3)	0 (0.0)	1 (6.7)	0 (0.0)
Synovial fluid	9	0 (0.0)	2 (22.2)	0 (0.0)	0 (0.0)	0 (0.0)
CSF	5	0 (0.0)	0 (0.0)	0 (0.0)	0 (0.0)	0 (0.0)
Aspirate	34	1 (2.9)	2 (5.9)	1 (2.9)	0 (0.0)	0 (0.0)
Breast aspirate	2	0 (0.0)	1 (50)	0 (0.0)	0 (0.0)	0 (0.0)
Gastric lavage	24	0 (0.0)	1 (4.2)	1 (4.1)	0 (0.0)	0 (0.0)
Total	251	7	14	12	2	1

Abbreviations: CSF, cerebrospinal fluid; FM, fluorescence microscopy; GNR, Gram‐Negative rods; GPC, Gram‐positive Cocci; GPR, Gram‐positive rods; ZN, Ziehl‐Neelsen.

**TABLE 3:** Specimen type and line probe assay (LiPA) results from extrapulmonary tuberculosis (EPTB) suspected patients.

Specimen type	Number	Positive molecular *n* (%)
Ascitic fluid	80	3 (1.99)
Pleural fluid	25	0
Pus	7	0
Synovial fluid	8	0
CSF	4	0
Aspirate	24	1 (0.66)
Breast aspirate	2	0
Gastric lavage	1	0
**Total**	151	4 (2.65)

Abbreviation: CSF, cerebrospinal fluid.

## DISCUSSION

TB remains a major public health challenge globally. Almost 20%–25% of all reported cases of TB are of extrapulmonary form. Early detection and drug susceptibility testing to identify resistant strains of MTBC are essential for efficient management and control of TB infection within communities. Diagnosis of EPTB with conventional methods is often associated with difficulties. This is because EPTB specimens are associated with irregular distributed paucibacillary load [19, 20]. The WHO recommends the use of rapid molecular diagnostic methods that have high diagnostic accuracy to help improve the early detection of the MTBC and identify drug‐resistant strains [1].

Ghana has made significant progress in improving TB case detection in the communities. Gene Xpert machines have been deployed round the country into selected facilities to improve TB detection particularly among PLHIV and early detection of drug‐resistant TB cases. Currently, Gene Xpert assay is used as an upfront test for TB diagnosis in Ghana. The assay detects MBTC and mutations causing resistance against Rifampicin but not isoniazid. The WHO recommends the use of LiPA as the initial test for the detection of isoniazid‐resistant TB (Hr‐TB) instead of phenotypic drug susceptibility testing (DST) among smear‐positive patients [1]. The LiPA further complements the Xpert MTB/RIF screening assay by validating rifampicin susceptibility and providing information on isoniazid susceptibility [21]. Reports further suggest that LiPA has a better diagnostic performance characteristic compared to GeneXpert when using smear microscopy as the gold standard [22, 23, 24], or the phenotypic proportion method on Lowenstein–Jensen media as the gold standard [25]. We applied the LiPA to analyse clinical specimens obtained from suspected EPTB patients reporting to the TTH to determine the prevalence of MTBC strains and their resistance to isoniazid and rifampicin.

The proportion of EPTB observed in the current study was (2.6%). This proportion is lower relative to the countrywide data (8%–10%) reported for Ghana [26] and the 21.8% proportion observed by Ohene et al. [6] in the Greater Accra region of Ghana. The observed proportion is again lower than rates reported in other African countries such as Swaziland (18.4%), Cameroon (19.4%) and Botswana (25%) [26]. The low proportion observed may be attributed to the number and the category of patients. The number of patients used in the current study was relatively low compared with the other studies. It is more likely to obtain higher positivity rate in the study with higher sample size. The rate of EPTB is generally higher among patients referred to the HIV clinics and PLWHIV [6]. The current study did not record patients from the HIV clinic or ward. Again, the performance characteristic of a test method is also dependent on the bacillary load in the specimen. This suggests that there may be specimens with a number of bacilli below the detection limit of the LiPA resulting in a low positivity rate.

The peritoneal cavity was the most common site (2.6%) of EPTB infection in the current study. The prevalence of EPTB infection relative to anatomic sites varies with geographic location and the socioeconomic status of the population. Several studies have reported varied anatomic sites such as pleural [27], genitourinary, meningeal [28] and lymph node as the most common site of infection [12, 29–31]. Ohene et al. reported disseminated TB and the pleura as the commonly affected extrapulmonary sites in the Greater Accra region [6]. Peritoneal TB is characterized by a slowly progressive abdominal swelling from ascites and abdominal pain and may appear because of the reactivation of latent infection from contaminated dairy products or dissemination via haematogenous spread from a primary lung focus, active pulmonary or miliary TB, or through lymph channels from infected nodes. Northern Ghana is home to approximately 80% of Ghana's livestock production, employing young adult smallholder farmers in urban and peri‐urban communities [32, 33]. There are abattoirs in all the regional capitals which receive livestock from the animal markets and other West African countries. Abattoir staff, including butchers, veterinary officers, sanitary inspectors and meat and livestock handlers in the region, sometimes work without the appropriate protective equipment. Milk and milk products sold in the markets sometimes come from uncertified farms where milk producers sold raw milk to intermediaries or directly to consumers. Livestock slaughtered at unapproved satellite slaughtering slabs and not certified by veterinary officers is sometimes sold at the markets. Consumption of unpasteurized milk and milk products and uncertified meat products is not uncommon in the region [34, 35, 36]. Control of TB in the region will require effective collaboration between veterinary and biomedical workers to implement public and animal health measures that will help curb TB transmission in the communities.

The current study observed a male to female ratio of 1:1 among the four EPTB infected patients. However, varied reports concerning the demographics of patients and the risk of EPTB infection have been documented. Although other studies have reported female dominance [4, 11, 37], Gunal et al. and Yang et al. have reported more males with EPTB [3, 28]. The current study indicated that EPTB was highest among patients aged 15–46 years. These results corroborate with previous studies [11, 12, 30]. Age is an important determinant for TB infection in high burden countries, and the associations between TB and men in their economically active (31–40) years have been highlighted [38, 39].

This study further observed that MTBC detection rate was higher with LiPA than microscopy. This is expected because smear microscopic methods, even though quick and cost effective, are less sensitivity [19], and in extrapulmonary specimens where mycobacteria are most often paucibacillary, the sensitivity of microscopy is even less [40]. Interestingly, the smear‐positive specimens were LiPA negative. This finding may be attributable to the fact that microscopy lacks the ability to differentiate members of MTBC from the nontuberculous mycobacteria. It is possible therefore that the smear‐positive bacilli were not members of the MTBC which were specifically detected by the LiPA. The detection of nontuberculous mycobacterium (NTM) by smear microscopy in clinical specimens is worth noting. The proportion of mycobacterial diseases caused by NTM is increasing steadily globally and has become a public health concern [41, 42, 43]. The NTM are emerging as important opportunistic pathogens responsible for significant morbidity and mortality in both immune competent and immune compromised individuals [41]. They are large and diverse groups of environmental mycobacteria associated with a variety of diseases in humans and mainly affect the lung, skin and soft tissue, lymph nodes and disseminated disease in patients with severely compromised immune system [43, 44]. Most of the NTM species are inherently resistant to anti‐TB agents, which makes the treatment of NTM infections more challenging. In resource‐limited communities where there are inadequate laboratory facilities to identify NTM to the species level, the combination of the molecular and the conventional methods is recommended [41, 45].

The current study recorded one MDR‐TB (resistance to isoniazid and rifampicin) and one mono isoniazid‐resistant TB (Hr‐TB). Isoniazid and rifampicin are two important first‐line antimycobacterial drugs, and resistance to these two drugs poses a serious threat to TB management and control [46]. The rapid drug susceptibility testing to detect drug‐resistant TB and prompt initiation of effective treatment regimen is crucial for TB control [47]. In countries where the Xpert MTB/RIF screening assay is offered as an upfront test for TB diagnosis, rifampicin resistance (RR) is used as a proxy for MDR‐TB. Therefore, drug‐susceptibility testing for Hr‐TB is not generally done [48]. Meanwhile, our finding corroborates with other reports that suggested that Hr‐TB cases are increasing globally and much more common than RR‐TB in resource‐limited communities [46, 49]. The disregard for drug‐susceptibility testing for isoniazid and Hr‐TB may lead to serious management challenges in the near future including increasing the risk of treatment failure or relapse and a greater probability of acquiring other drug‐resistant TB [46, 49]. Furthermore, it may lead to the ineffectiveness of isoniazid preventive therapy which refers to the use of isoniazid to treat latently infected TB patients [50, 51].

The absences of WT2 and WT3 *rpo*B bands for RIF and WT1 and WT2 *inh*A bands for INH were demonstrated by the MDR strain. The INH mono‐resistant strain demonstrated the absence of *inh*A WT1 and *inh*A WT2 bands. Contrary to the current study, several studies have reported different rates of drug‐resistant MTBC with mutations in the *rpoB* gene of RIF and *katG* gene of INH conferring resistance to MTBC strains in extrapulmonary specimens [27, 52, 53]. Isolates habouring mutation in *katG* gene expresses high‐level INH resistance and are associated with unfavourable treatment outcome such as patient relapse and death [45, 54]. Mutation in *inhA* gene is associated with low‐level INH resistance [55]. Despite the intriguing findings observed in the current study, it has some limitations. These include relatively low samples analysed, incomplete laboratory records on specimens and lack of detail patients’ clinical characteristics. This may have introduced an element of bias in the results. Therefore, the result should be interpreted with caution.

## CONCLUSION

The current study has shown that EPTB is a public health problem in the region, with the peritoneal cavity being the most common site of infection. It has also confirmed the limitation of only microscopy for the detection and diagnosis of EPTB. It is therefore imperative to strategically resource selected health facilities across the region with advanced molecular technologies to enhance TB surveillance especially EPTB.

## AUTHOR CONTRIBUTIONS

Yaa Nyarko Addai and Samuel E. K. Acquah developed the concept of the study. Yaa Nyarko Addai and David Zeyeh retrieved the stored samples and all required data. Yaa Nyarko Addai, David Zeyeh and Honesty Mensah Ganu processed the samples. Samuel E. K. Acquah, Yaa Nyarko Addai and Ezekiel Kofi Vicar drafted the manuscript with significant contributions from Abass Abdul Karim, Walana Williams, Israel Mensah Attipoe and Lawrence Quaye. All authors have read and approved the final manuscript.

## CONFLICT OF INTEREST STATEMENT

The authors declare that they have no conflicts of interest.

## FUNDING INFORMATION

The authors received no specific funding for this work.

## ETHICS STATEMENT

Approval for the conduct of the study was obtained from the Ethical and Research Committee of the University for Development Studies (UDS/RB/0121/22).

## TRANSPARENCY STATEMENT

The lead author affirms that this manuscript is an honest, accurate and transparent account of the study being reported; that no important aspects of the study have been omitted; and that any discrepancies from the study as planned (and, if relevant, registered) have been explained.

## Data Availability

The data set for this study is available with the corresponding author and will be made available upon reasonable request. The corresponding author has full access to all the data in this study and takes complete responsibility for the integrity of the data and the accuracy of the data analysis.
